# The Effect of COVID-19 Lockdown Measures on Physical Activity Levels and Sedentary Behaviour in a Relatively Young Population Living in Kosovo

**DOI:** 10.3390/jcm10040763

**Published:** 2021-02-14

**Authors:** Masar Gjaka, Kaltrina Feka, Antonino Bianco, Faton Tishukaj, Valerio Giustino, Anna Maria Parroco, Antonio Palma, Giuseppe Battaglia

**Affiliations:** 1 Department of Sport and Movement Science, University for Business and Technology, 10000 Pristina, Kosovo; masar.gjaka@ubt-uni.net; 2 Department of Psychology, Educational Science and Human Movement, University of Palermo, 90128 Palermo, Italy; kaltrina.feka@unipa.it (K.F.); antonino.bianco@unipa.it (A.B.); valerio.giustino@unipa.it (V.G.); annamaria.parroco@unipa.it (A.M.P.); antonio.palma@unipa.it (A.P.); 3 Sport and Exercise Science Research Unit, University of Palermo, 90144 Palermo, Italy; 4 PhD Program in Health Promotion and Cognitive Sciences, University of Palermo, 90128 Palermo, Italy; 5 Faculty of Physical Education and Sport, University of Pristina, 10000 Pristina, Kosovo; faton.tishukaj@uni-pr.edu; 6 Regional Sports School of CONI Sicilia, 90141 Sicily, Italy

**Keywords:** physical activity, COVID-19, Kosovo, restrictions, public health

## Abstract

To abate the spread of the COVID-19 virus, different restriction measures were imperative, limiting the possibility to be engaged in physical activity. Therefore, this study aimed to evaluate the effect of COVID-19 lockdown on physical activity (PA) levels expressed as energy expenditure (MET-min/week) and sedentary behaviour in Kosovo. The possible association between PA levels and other factors was analyzed. 1633 participants (age range: 13 to 63 years; mean: 24.70 ± 9.33 years; body height: 172 ± 10.57 cm; body mass: 69.10 ± 13.80 kg; BMI: 23.09 ± 3.63 kg/m^2^) participated in the study, categorized by age, gender, BMI, and living area. An online survey, including an adapted version of the IPAQ-SF, was administered once during lockdown to assess PA levels and sedentary behaviour both before and during COVID-19 lockdown. The Wilcoxon signed-rank, Mann–Whitney U and Kruskal–Wallis rank of sum tests were used for statistical analysis. COVID-19 restrictions had a negative impact on the types of and overall PA levels MET-min/week (*p* < 0.001). Sedentary behaviour significantly increased during COVID-19 restrictions (*p* < 0.001). Higher decreases in MET-min/week during lockdown were observed among males, young and young adults, overweight, and urban-living participants. Finally, COVID-19 restrictions decreased the PA levels and MET-min/week, and increased sedentary behaviour also in a relatively young cohort. Such differences were dependent on several factors.

## 1. Introduction

In the last decades, the literature has extensively described the numerous benefits of physical activity (PA) on health, including aspects directly associated with the amelioration of general health as well as the prevention of cardiovascular diseases, type II diabetes, colon cancer, immune function, and obesity [1,2,3,4]. The ability of different types of exercise to respond also to bone, muscle, and joint pathologies as well as degenerative diseases has been very well established [5,6,7,8,9,10].

Conversely, sedentary behaviour and a lack of daily PA might lead to health-related problems such as dyslipidaemia [11], microvascular dysfunction, and problems associated with peripheral insulin resistance [12], which are related to increased odds of weight gain and an accompanying increased risk of indicators for cardiometabolic health-related risks [13]. Studies regarding health-related adverse effects of physical inactivity have defined it as the fourth leading risk factor, accounting for 6% of global deaths [14]. 

Since the beginning of 2020, humankind is globally fighting against a deadly virus (COVID-19) which is a very serious public health concern, initially detected in Wuhan (China) at the end of December 2019 [15,16]; it is now spread out worldwide, having infected over 48 million people and causing over 1.2 million deaths to date [17]. Due to its fast human-to-human transmission and the damage the virus causes to human beings—in serious cases, resulting in death—the World Health Organization (WHO) has declared the COVID-19 virus a pandemic (11 March 2020) [18]. In view of all the facts mentioned above, the WHO and other relevant national bodies, including also their respective governments, have implemented different kinds of lockdown strategies relative to the severity of the COVID-19 outbreak [19] including isolation, home confinement, and also social distancing [20]. In addition, due to the precaution measures imposed by the respective countries, restricted access to PA spaces (e.g., gyms) has limited PA possibilities, especially for activities usually performed outdoors and/or in groups [19].

After the first COVID-19 positive case was detected (March 13), the precaution measures were also taken in Kosovo by closing universities, schools, and borders, and also by suspending social gatherings and sports competitions at amateur and professional levels [21,22].

Yet, although social distancing and self-isolation are acknowledged as effective measures to flatten the spreading curve of COVID-19, these measures are also considered to increase the burden among humans [23,24]. In fact, home confinement and other imposed safety measures have negatively influenced PA levels, which has already been reported in other studies in countries such as Italy [25], Croatia [26], Spain [27], Canada [28], and also in large-scale surveys including countries from all over the world [20]. Furthermore, the negative impact of prolonged isolation on psychological well-being, causing post-traumatic stress symptoms, confusion, and anxiety, has been reported [29]. Moreover, studies have shown an increased time spent in sedentary behaviour (sitting time) during the COVID-19 pandemic compared to pre-pandemic time [20], which might be an increased concern for public health. Indeed, Hossain and his colleagues (2020) highlighted that there might be an association between regional anti-COVID-19 policies and socio-economic factors with differences in PA levels during COVID-19 lockdown [30]. Additionally, COVID-19 lockdown consequences on behavioural outcomes, including PA, affect all inhabitants regardless of age, sex, and ethnicity [10]. On the contrary, the living area was found to be an important factor related to the PA levels during COVID-19 lockdown [31].

In light of these facts, besides noticeable and severe changes, to the best of the authors’ knowledge, no scientific data have been reported regarding the effect of COVID-19 lockdown measures on PA levels and sedentary behaviour in the Kosovo population. 

Therefore, this exploratory study aimed to evaluate the effect of COVID-19 lockdown in PA levels expressed as energy expenditure (MET-minutes/week) and sedentary behaviour among the Kosovo population. Additionally, the potential link between factors such as age, gender, anthropometrics, and living area and the level and frequency of PA has been investigated.

We hypothesized that the COVID-19 lockdown would have a negative impact on the Kosovan population by decreasing the PA level and increasing the exposure time to sedentary behaviour. Additionally, it is hypothesized that the PA level and MET-minutes/week changes during COVID-19 lockdown depend on age, gender, anthropometrics, and living area.

## 2. Materials and Methods

### 2.1. Study Design and Procedure

The current study is a cross-sectional study design implemented using an online survey, including an adapted version of the International Physical Activity Questionnaire- Short Form (IPAQ-SF), created on the Google Forms platform (Google LLC, Mountain View, CA, USA) during the COVID-19 pandemic outbreak in Kosovo. During the period this study was conducted, Kosovo inhabitants were exposed to the government anti-COVID-19 restriction measures.

Approximately four weeks after the lockdown measures started (13 March 2020), the online survey was launched and randomly dispersed to as many people as possible. Initially, an official email including the link of the online survey was sent to all students and academic and non-academic staff of the University for Business and Technology. Afterwards, in order to increase the number of participants, the snowball sampling recruitment approach was used, spreading the online survey link via e-mailing channels and social media such as Instagram, Facebook, WhatsApp and Viber, encouraging participants to enroll in the study [20,25,27,28,32].

The study was conducted in accordance with the principles of the Declaration of Helsinki and received ethical clearance from the Ethics Committees of the University of Palermo (Protocol Number: 14/2020).

### 2.2. Participants

Participants who completed the online survey launched on 8 April until 21 April 2020 (during the government restriction measures) were considered eligible for the current study. 

An overall total of 1928 of participants representing the country participated in the study by filling out the online survey. In order to minimize the effect of errors, a data cleaning process was adopted comprised of the following steps: exclusion of ineligible respondents including participants who did not complete the entire survey and of multiple submissions from the same participant; identification and management of meaningless data (e.g., duration expressed in hours, not in minutes; duration reported as weekly, not daily; duration reported as text, not as a number etc.). 

Yet, after the cleaning process, a total of 1633 participants were included in the study.

### 2.3. Internal Consistency of the Questionnaire 

The questionnaire used within the current study was built from IPAQ-SF. The questionnaire focused on two different moments, relating to before and during COVID-19 confinement restrictions. Specifically, it consisted of 31 items, assessing demographics (question 2 and 3), anthropometrics (question 4 and 5), PA before COVID-19 (question 7), employment status and residence during COVID-19 confinement (questions 8 to 13), the frequency and duration of vigorous-intensity PA before and during COVID-19 confinement (questions 14 to 17), the frequency and duration of moderate-intensity PA before and during COVID-19 confinement (questions 18 to 21), the frequency and duration of walking before and during COVID-19 confinement (questions 22 to 25), sedentary behaviour before and during COVID-19 confinement (question 26 and 27), and information related to PA during COVID-19 confinement. The introductory page of the questionnaire provided a concise description of the study and its main purposes, as well as the declarations of anonymity and confidentiality, and the personal data protection policy.

Since the questionnaire collected information of two different moments, to assess the validity of the obtained answers relating to past behaviour, question 7—“Before COVID-19 quarantine, how many days/week did you train regularly?” (with options ranging from 0 to 7 days per week)—was categorized into a dichotomous variable; according to the American College of Sports Medicine (ACSM) physical activity guidelines, people are considered sedentary if they train from 0 to 2 days per week, while they are considered physically active if they train from 3 to 7 days per week [33]. Therefore, a new variable, “physically active”, with possible answers “Yes” or “No” was derived from the previous question.

Afterwards, to check if people where honest in their answers, a Mann–Whitney test was performed comparing the level of PA intensity (vigorous, moderate, walking) between sedentary and physically active individuals before the quarantine. If the respondents were honest in their answers, and the questionnaire was sensitive in detecting the amount of PA prior to the COVID-19 lockdown, sedentary people should also have reported less pre-confinement PA than active people. From the results reported in Table 1, sedentary respondents reported an amount of the three intensities of PA that is significantly lower than physically active respondents, indicating that they were honest in indicating their PA before quarantine.

### 2.4. Scoring Protocol

Based on the well-known concept of metabolic equivalent (MET), the total weekly PA level, expressed as energy expenditure (MET-min/week), was calculated [34]. Consequently, the total weekly energy expenditure (MET-min/week) was estimated according to the specific metabolic equivalent (MET) values for each category of PA (3.3 MET for walking, 4 MET for moderate-intensity PA, and 8 MET for vigorous-intensity PA) [35,36,37]. The following formula was used to calculate the total weekly energy expenditure (MET-min/week): *total weekly energy expenditure (MET-min/week) = MET x duration of PA type (minutes) x frequency.* All calculations were performed in accordance with the specifications provided elsewhere [35,36,37], and also following the “Guidelines for Data Processing and Analysis of the International Physical Activity Questionnaire (IPAQ)—Short and Long Forms” (http://www.ipaq.ki.se (accessed on 15 September 2020)) [38].

Furthermore, following the IPAQ guidelines for scoring protocol (http://www.ipaq.ki.se (accessed on 15 September 2020)) [38], according to the total weekly energy expenditure (MET-min/week) from all types of PA, participants were assigned to one of the following categories: (1) low activity (<600 MET-min/week), (2) moderate activity (≥600 MET-min/week), and (3) high activity (≥3000 MET-min/week).

### 2.5. Statistical Analysis

Several variables derived from the online survey were re-coded for further statistical analyses. The following categories were used to categorize participants based on their body mass index (BMI) levels: underweight (UW) (BMI < 18.5), normal weight (NW) (18.5 < BMI < 25), and overweight (OW) (BMI > 25) [25,39]. Participants were grouped into five different age categories: young (≤ 25 years), young adults (25 < years ≤ 35), adults (35 < years ≤ 55), senior adults (55 < years ≤ 65), and elderly (>65 years) [25,40]. The Shapiro–Wilk test was used to test the normality of the distribution for all variables. Data are computed and presented as means, standard deviation, and percentages. 

To test the difference in the total energy expenditure (MET-min/week) before and during COVID-19 confinement, the Wilcoxon signed-rank test for dependent groups was used. The Mann–Whitney U test was chosen to analyze the differences in responses before and during for type of PA, gender, and living area (urban vs. rural living environment). To assess pre- and during COVID-19 confinement differences between categories assigned based on BMI and age, the Kruskal–Wallis rank-sum test was employed, with the Mann–Whitney U test chosen for pairwise comparisons. Non-parametric tests have been used because the normality assumption of the distribution, tested by the Shapiro–Wilks test, was violated.

The level of significance was set at *p* < 0.05. All of the statistical analyses were performed using the Statistical Package for Social Sciences (SPSS), version 21.0 (SPSS Inc., Chicago, IL, USA). GraphPad Prism version 8.4.3 was used to design graphs and figures.

## 3. Results

### 3.1. Descriptive Analysis

#### Participants

A total of 1633 (812 males and 823 females) Kosovan participants (age range: 13 to 63 years; mean: 24.70 ± 9.33 years; body height: 172 ± 10.57 cm; body mass: 69.10 ± 13.80 kg; BMI: 23.09 ± 3.63 kg/m^2^) who all voluntarily participated in the study. All of the demographic characteristics of participants including gender, age, BMI category, and living area are presented in Table 2.

### 3.2. Physical Activity before and during the COVID-19 Confinement Period

All PA results derived from the questionnaire, registered before and during home confinement, are presented in detail in Table 3.

#### 3.2.1. Vigorous Intensity Physical Activity

The number of days/week and minutes/day of vigorous intensity PA during compared to before home confinement declined significantly by 25.7% (*p* < 0.001) and 36.2% (*p* < 0.001), respectively. Moreover, the energy expenditure (MET-min/week) of vigorous intensity PA decreased by 45.7% during compared to before home confinement (*p* < 0.001).

#### 3.2.2. Moderate Intensity Physical Activity

A statistically significant reduction in the number of days/week (17.1%) and minutes/week (27.5%) of moderate intensity PA during home confinement was found. Likewise, significantly lower MET-min/week values were found during compared to before home confinement (36.4%; *p* < 0.001).

#### 3.2.3. Walking

The number of days/week of walking declined significantly by 28.5% during home confinement (*p* < 0.001). Similarly, the number of minutes/week and the MET-min/week of walking significantly decreased (*p* < 0.001) by 41.3% and 52.9%, respectively.

#### 3.2.4. All Physical Activity

Likewise, the number of days/week, minutes/week, and MET-min/week of all PA was significantly lower during home confinement by 24.32%, 34.8%, and 26.2%, respectively. 

#### 3.2.5. Sitting Time

The statistical analysis indicated a significant increase in the number of sitting hours/day by 34% during home confinement (*p* < 0.001). 

**Figure 1:**
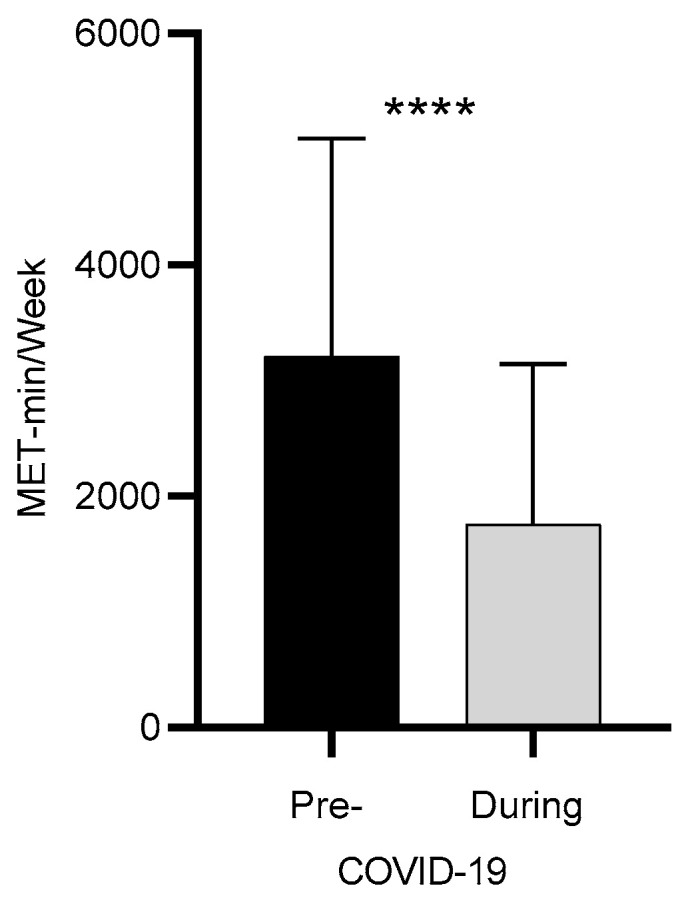
Overall MET-min/week (pre and during COVID-19). Total weekly energy expenditure (MET-min/week) pre-home confinement, during home confinement, and the difference between pre- and during home confinement. Legend: ****: (*p* < 0.001).

As depicted in Figure 1, significant differences were reported in the energy expenditure expressed as (MET-min/week) before and during home confinement (*p* < 0.001). 

Following the IPAQ recommendations for scoring protocol, participants were assigned to one of the three PA categories (i.e., < 600; ≥ 600; ≥ 3000 MET–min/week). For the pre-home confinement condition, results revealed that 49 participants belonged to the low activity category (3%), 801 were moderately active (49.1%), and 783 (47.9%) participants were highly active. In this regard, results for during the home confinement condition revealed an increase in the low activity category (*n* = 311; 19%) and in the moderate activity category (*n* = 1069; 65.5%), while a decline in the number of high activity participants was found (*n* = 253; 15.5%).

### 3.3. Energy Expenditure in Relation to Gender, Age Category, BMI Classification

**Figure 2:**
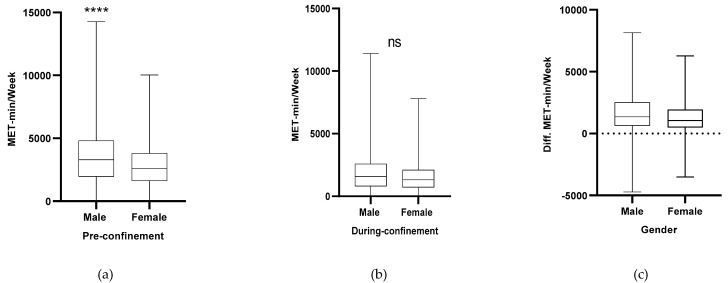
Gender differences in MET-min/week (pre and during COVID-19). (**a**) Total weekly energy expenditure (MET-min/week) pre home confinement condition in relation to gender category. (**b**) Total weekly energy expenditure (MET-min/week) during home confinement condition in relation to gender category. (**c**) Total weekly energy expenditure (MET-min/week) difference between pre and during home confinement in relation to gender category. Legend: ****: (*p* < 0.001); ns: non-significant difference.

Significant gender differences in MET-min/week were found (*p* < 0.001), with male participants expressing higher values of energy expenditure in the pre-confinement condition compared to the female participants (Figure 2a). On the other hand, no significant gender differences were found during the confinement condition in MET-min/week (*p* = 0.53) (Figure 2b). The difference between pre- and during confinement showed a higher decrease in MET-min/week among male participants (Figure 2c).

**Figure 3:**
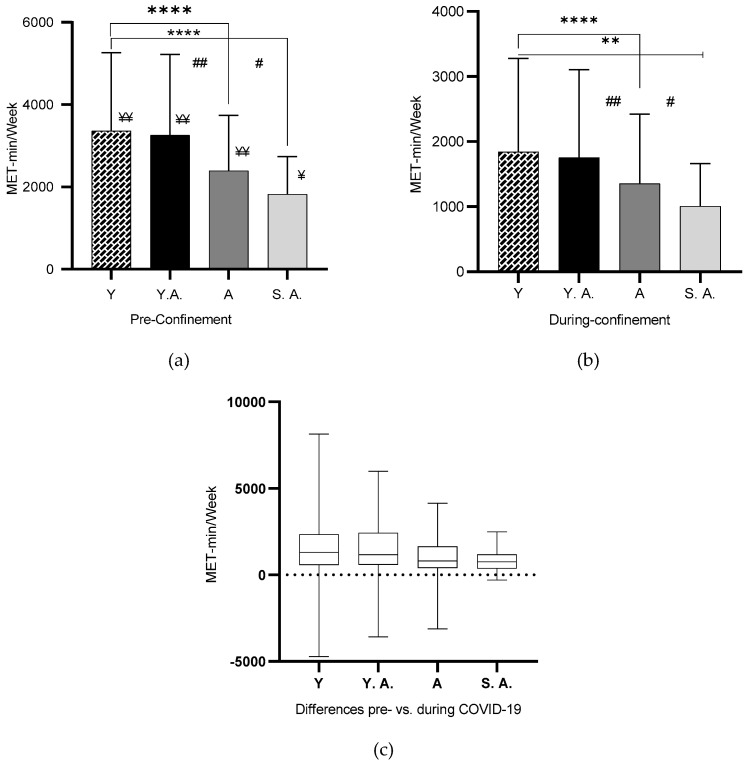
Age category differences in MET-min/week (pre and during COVID-19). (**a**) Total weekly energy expenditure (MET-min/week) pre-home confinement condition in relation to age categories. Legend: Y: young; Y. A.: young adult; A: adult; S. A: senior adult; ****: (*p* < 0.001); ##: (*p* < 0.001); #: (*p* = 0.002); ¥¥: differences between pre and during confinement (*p* < 0.001); ¥: differences between pre- and during confinement (*p* = 0.006). (**b**) Total weekly energy expenditure (MET-min/week) during home confinement condition in relation to age categories. Legend: Y: young; Y. A.: young adult; A: adult; S. A.: senior adult; ****: (*p* < 0.001); **: (*p* = 0.005); ##: (*p* = 0.001); #: (*p* = 0.02). (**c**) Total weekly energy expenditure (MET-min/week) difference between the pre- and during-confinement condition in relation to age categories. Legend: Y: young; Y. A.: young adult; A: adult; S. A.: senior adult.

Analysis of the age categories showed a significant difference in MET-min/week between pre and during home confinement condition (*p* < 0.001), except for senior adults, where the difference was (*p* = 0.006). Pairwise comparison analysis revealed a significant difference between the young and adults (*p* < 0.001), young and senior adults (*p* < 0.001), young adults and adults (*p* < 0.001), and young adults and senior adults (*p* = 0.002) in MET-min/week pre confinement condition (Figure 3a). Likewise, the during-confinement condition pairwise analysis showed the same significant differences between the young and adults (*p* < 0.001), young and senior adults (*p* = 0.005), young adults and adults (*p* = 0.001), and young adults and senior adults (*p* = 0.02) in MET-min/week for the during-confinement condition (Figure 3b). As depicted in Figure 3c, the highest MET-min/week difference between pre- and during confinement was found among the young and young adult categories.

**Figure 4:**
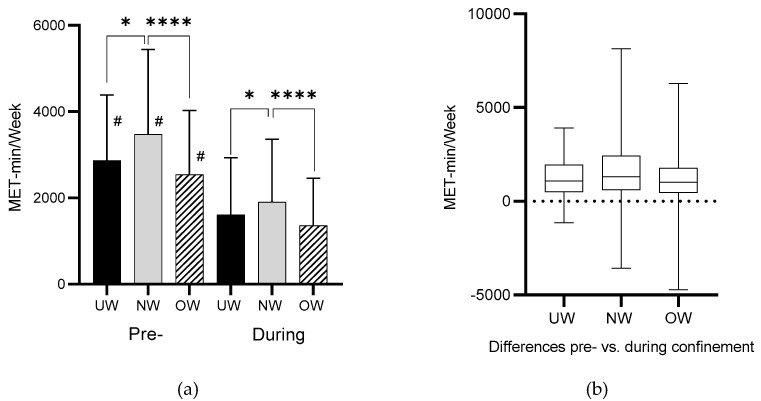
BMI category differences in MET-min/week (pre and during COVID-19). (**a**) Total weekly energy expenditure (MET-min/week) pre and during home confinement condition in relation to BMI category. (**b**) Total weekly energy expenditure (MET-min/week) difference between pre and during home confinement in relation to BMI category. Legend: UW—underweight; NW—normal weight; OW—overweight; *—(*p* < 0.05); ****—(*p* < 0.001); #—(*p* < 0.001).

Analysis regarding the differences between BMI categories revealed that during the pre-confinement condition the NW category showed a higher MET-min/week value compared to the UW (*p* = 0.006) and OW (*p* < 0.001) categories (Figure 4a). Likewise, in the during-confinement condition, the NW category showed a higher MET-min/week value compared to both UW and OW (*p* = 0.04) and (*p* < 0.001), respectively. On the other hand, when comparing MET-min/week between pre- and during-confinement conditions, statistically significant differences were found for all BMI categories (*p* < 0.001). Moreover, the analysis of MET-min/week differences between the pre- and during-confinement condition revealed that the NW was the category with the highest difference (Figure 4b).

### 3.4. Urban vs. Rural Living Environment

**Figure 5:**
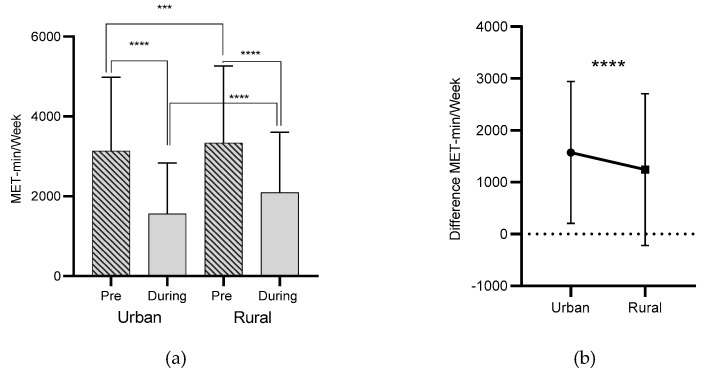
Living environment differences in MET-min/week (pre and during COVID-19). (**a**) Total weekly energy expenditure (MET-min/week) pre- and during home confinement in relation to urban and rural living environment. (**b**) Total weekly energy expenditure (MET-min/week) difference between pre- and during home confinement in relation to urban and rural living environment. Legend: ****: (*p* < 0.001); ***: (*p* = 0.002).

Statistical analysis showed significant differences in MET/min/week for the pre-confinement condition between participants living in urban and rural environments (*p* = 0.002). Additionally, significant differences in the total weekly energy expenditure (MET-min/week) were depicted for the during-confinement condition (*p* < 0.001), with participants living in rural environments reporting higher values (Figure 5a). On the other hand, the analysis revealed significant changes in MET-min/week between the pre- and during-confinement condition, for both participants living in urban and rural environments (*p* < 0.001) and (*p* < 0.001), respectively (Figure 5a). Likewise, significant differences were recorded in the MET-min/week difference between urban and rural living environments, with a higher decrease among urban living participants (*p* < 0.001) (Figure 5b).

## 4. Discussion

The purpose of this study was to evaluate the effect of COVID-19 lockdown in PA levels expressed as energy expenditure (MET-minutes/week) and sedentary behaviour among the Kosovo population. Additionally, this study analysed the possible effect of covariates such as age, gender, anthropometrics, and living area on the PA levels of the Kosovan population. 

This study has provided several important findings. The main finding of the present study showed a negative effect of COVID-19 home confinement on PA levels, leading to a significant decrease in days, minutes/day, and energy expenditure (MET-minutes/week) for three types of PA (vigorous intensity, moderate intensity, and walking activity) compared to the pre-home-confinement condition, as well as on overall PA level expressed as energy expenditure (MET-minutes/week).

Similarly, decreased PA levels during home confinement were reported also in other similar recent studies in people with and without disabilities [20,25,26,27,28,41,42,43]. Unsurprisingly, current results have demonstrated a significant negative effect of home confinement on PA intensity, causing an increased proportion of participants in the low and moderate activity category (16% and 17.6%, respectively), and a decrease in the high activity cohort of 32.4%. Further, this study strengthens the findings reported in comparable recent research, in which the authors reported the same pattern of PA reduction in active Sicilian adults due to home confinement restrictions [25]. 

As previously reported, sport clubs play a massive role in maintaining higher PA levels [44]. Particularly for Kosovo, the proportion of young individuals doing sports or other types of PA is relatively high as compared to older ones. Taking this into consideration, closing gyms, sports clubs and other public and private spaces has had the most detrimental impact on PA levels in the majority of the Kosovo population, taking into account the population demography. In addition, the lack of technical knowledge of the population on appropriate training routines has been reported as a factor leading to lower PA levels [45]. While other countries during COVID-19 lockdown were encouraging and stimulating their inhabitants to get enrolled in non-intensive indoor and outdoor PA to stimulate their mental and physical well-being [46], in Kosovo, no such governmental campaigns were organized. 

Sitting behaviour (hours/day) was significantly higher during home confinement compared to pre-home-confinement. Results indicated a 34% increase in sitting behaviour (hours/day) during home confinement, most likely because participants were ordered to stay inside their homes to avoid infection. These results are in accordance with results reported in a recent large-scale research representing a multi-national and multi-continental sample, where a significant increase in sitting behaviour during home confinement was reported [20].

The amount of decrease in the PA level might be affected by different factors. In this regard, when our results were stratified by gender, significant differences were found between genders in PA levels pre-home-confinement, but such differences were not present during COVID-19 home confinement. A decrease in PA levels due to COVID-19 home confinement was noticed among both genders, with a higher drop among males. These findings are not in accordance with the results observed in a previous research, where gender differences in PA level during home confinement were reported [25]. This fact could be explained by the cultural context in which Kosovan people live in. In general, females living in Kosovo are more involved in household activities compared to males, which has helped the female participants to prevent as much of a decrease in PA levels as among males. This fact is supported by scientific evidence where household chores were reported to be the most prevalent PA type during home confinement [27]. 

For the age category variable, our results have revealed significant differences between pre- and during COVID-19 home confinement in PA levels. The highest PA level for both pre- and during home confinement conditions was reported from the young and young adult categories, while senior adults reported the lowest PA level. These findings are in complete agreement with the results published showing a similar trend of PA levels pre- and during home confinement [25,47]. Indeed, it has been previously reported that younger people are more physically active than the older ones [48] and the PA level progressively decreases with aging [43]. These results could have possibly been affected by such a high number of young and young adult participants. Nevertheless, this high number of young and young adult participants could be considered as representative for Kosovo, since the majority of the people belong to these two categories [49]. Considering the increased mortality rate of COVID-19 among older individuals, it might be speculated that this has prevented them from moving outside their living environments, resulting in higher levels of inactivity. Nevertheless, a recent study has shown that older adults who met the WHO physical activity guidelines during home confinement had better mental well-being [23]. Bearing this in mind, despite the restrictions, it is recommended that older adults engage in physical activity, even in their houses.

Furthermore, results of the current study have revealed significant differences in energy expenditure (MET-min/week) between BMI categories, with, as generally expected, the normal weight category expressing the highest values before and during COVID-19 home confinement. In fact, significant differences in energy expenditure between pre- and during home confinement existed for all three categories (underweight, normal weight, and overweight), with the normal weight category reporting the highest MET-min/week difference between pre- and during COVID-19 home confinement. This is not supported by the results of a similar study recently published, where no significant differences between BMI categories in energy expenditure were reported pre-confinement, but differences were reported to be present among the overweight category during COVID-19 home confinement [25]. Indeed, a higher decrease of PA during home confinement has been reported among participants with lower BMI compared to their counterparts with higher BMI [50].

Living area has been reported as an important factor influencing PA levels and fitness [51,52,53]. In this regard, our results demonstrated that the decrease in PA levels of Kosovan people during lockdown living in rural areas was smaller compared to the urban living ones, which showed a larger decrease in their PA levels. Such differences could be explained by the fact that people who live in rural areas of Kosovo usually have plenty of outdoor spaces and most of them are involved in farming and householding activities, which might have helped them to preserve their PA levels during the COVID-19 restrictions. These results are in line with the findings reported for Croatian adolescents, in which the authors found a larger decrease in PA levels during lockdown among urban living adolescents compared to their rural living counterparts [31].

### Strengths and Limitations of the Study

To the best of our knowledge, this is the first study with a relatively large number of participants to assess the impact of the COVID-19 pandemic and associated public health restrictions on the PA behaviour of Kosovan population. 

As a strength, it is worth noting that, besides having a representative sample form the entire country, in this study, the participants’ living environment was also included. Taking into consideration the importance of PA and the maintenance of its levels during COVID-19 home confinement, we think that these results could be of importance to plan and develop proper strategies and policies when facing situations such as a pandemic.

Beside its strengths, this study also had some limitations that should be acknowledged. PA was not objectively measured, but was assessed by a self-reported questionnaire, increasing the risk of self-reporting bias. Nevertheless, since the questionnaire was administered both before and during COVID-19 home confinement, this bias does not influence our results to a great extent. 

Another limitation is the comparability with the other studies, which, for their purposes, have used different methodologies and instruments. 

A limitation that must be highlighted is the fact that it was not possible to demonstrate the validity of the questionnaire through the common analysis methods (i.e., using objective measures of PA from movement devices such as accelerometers or pedometers for comparison with the self-reported measures) [54]. In fact, the common analysis methods used for the validation are:

(1) correlation of the self-reported PA data with data from a relative measure of an objective measurement device
(2) computation of the absolute differences between self-reported and objective measurements [54].

However, we were unable to perform either the first or second method due to government restrictive measures which limited the possibilities of PA and therefore the consequent objective measure of PA practiced.

However, although the instrument used has detected the PA levels related to two different periods (i.e., pre- and during COVID-19 confinement measures) in a single administration, it should be noted that:

(1) The IPAQ is an instrument allows to evaluate the physical activity levels for either the last seven days (in the present study, the period during COVID-19 confinement) or the usual week (that we can consider for the questions related to the pre-COVID-19 confinement) [35];
(2) The results related to the PA levels of the week before COVID-19 confinement were compared and resulted consistent with those already published in the literature [55,56];
(3) The items related to the PA levels of the week during COVID-19 confinement refer to the classic IPAQ procedure in which the level of PA of the previous seven days is assessed.

## 5. Conclusions

Finally, based on the results, it can be concluded that COVID-19 home-confinement measures have had a negative impact on PA levels and energy expenditure (MET-min/week), especially among males, overweight participants, and those participants living in an urban area. The results of this study indicated also a significant increase in sitting behaviour among the Kosovan population during the COVID-19 lockdown.

Moreover, significant differences between age categories in MET-min/week were detected, with higher MET-min/week values among young and young adult categories for both pre- and during COVID-19 home confinement conditions. A high number of participants in the young and young adult age categories might have affected the decreased amount of PA levels compared to the latter, which represents the real demographics of the Kosovo population. Nevertheless, MET-min/week values in these two categories were affected the most due to COVID-19 restrictions. The lower number of adult and older adult participants has prevented us from drawing general conclusions regarding the decrease of PA levels for the entire population. 

Currently, we are facing the second wave of COVID-19 infections, and these results could have potential important implications to help the decision-making bodies to implement strategies keeping in mind the maintenance of PA levels as crucial part of the public health sector.

## Figures and Tables

**Table 1:** Internal consistency of the questionnaire: types of PA before COVID-19 quarantine declared by sedentary and physically active respondents (According to the ACSM Physical Activity Guidelines 2018).

	Sedentary (*n* = 704)			Physically Active (*n* = 929)			
	Mean	SD	Median	Mean	SD	Median	*p*
**VPA**							
Days	1.40	1.52	1.00	3.41	1.64	4.00	<0.001
Minutes	26.19	30.09	20.00	53.37	30.86	60.00	<0.001
MET	533.35	685.96	240.00	1673.20	1195.64	1140.00	<0.001
**MPA**							
Days	3.20	2.02	3.00	4.07	1.66	4.00	<0.001
Minutes	50.65	36.86	45.00	64.62	35.13	60.00	<0.001
MET	986.27	727.92	792.00	1119.14	753.18	990.00	<0.001
**Walking**							
Days	5.32	1.79	5.00	5.70	1.60	6.00	<0.001
Minutes	53.63	32.53	45.00	57.79	33.33	60.00	0.01
MET	986.27	727.92	792.00	1119.14	753.18	990.00	<0.001

VPA: vigorous physical activity; MPA: moderate physical activity.

**Table 2:** Demographic characteristics of the participants.

Variables		N	%
**Sample**			
	Participants	1633	
	Female	823	50.4
	Male	810	49.6
**Age Categories**			
	Young	1130	69.2
	Young adults	299	18.3
	Adults	176	10.8
	Senior adults	28	1.7
**BMI Category**			
	Underweight	123	7.5
	Normal weight	1129	69.1
	Overweight	381	23.3
**Living Area**			
	Urban	1032	63.2
	Rural	601	36.8

N: number; %: percentage; BMI: Body Mass Index.

**Table 3:** Physical activity responses recorded pre- and during confinement.

		Pre-Confinement	During Confinement	Δ (Δ%)	*p* Value
**VPA**	Days/week	2.54 ± 1.87	1.89 ± 1.76	0.65 (25.7%)	<0.001
	min/week	41.7 ± 33.7	26.6 ± 25.3	15.09 (36.2%)	<0.001
	MET/week	1181.8 ± 1155.2	641.9 ± 794.4	539.94 (45.7%)	<0.001
**MPA**	Days/week	3.7 ± 1.9	3.1 ± 1.9	0.63 (17.1%)	<0.001
	min/week	58.6 ± 36.54	42.5 ± 29.6	16.11 (27.5%)	<0.001
	MET/week	967.3±765.4	615.5 ± 578	351.78 (36.4%)	<0.001
**Walking**	Days/week	5.54 ± 1.69	3.98 ± 2.44	1.58 (28.5%)	<0.001
	min/week	56 ± 33.04	32.9 ± 22.6	23.12 (41.3%)	<0.001
	MET/week	1061.9 ± 745	500.8 ± 478.9	561.1 (52.9%)	<0.001
**All PA**	Days/week	3.92 ± 1.23	2.96 ± 1.43	0.95 (24.32%)	<0.001
	min/week	52.1 ± 23.4	33.9 ± 18.7	18.11 (34.8%)	<0.001
	MET/week	3211 ± 1880	1756.5 ± 1387.2	493.45 (26.2%)	<0.001
**Sitting**	hours/week	5.33 ± 1.7	8.13 ± 2.2	2.8 (34%)	<0.001

VPA: vigorous physical activity; MPA: moderate physical activity; Δ: difference before vs. during confinement; Δ%: percentage of difference before vs. during confinement.

## Data Availability

The raw data will be made available by the authors, without undue reservations, upon request to the first author of this article.
